# Targeting miR-21 with NL101 blocks c-Myc/Mxd1 loop and inhibits the growth of B cell lymphoma

**DOI:** 10.7150/thno.53561

**Published:** 2021-01-19

**Authors:** Shu Li, Xin He, Yichao Gan, Jiawei Zhang, Feiqiong Gao, Limin Lin, Xi Qiu, Teng Yu, Xuzhao Zhang, Panpan Chen, Jiefeng Tong, Wenbin Qian, Yang Xu

**Affiliations:** 1 Department of Hematology, the Second Affiliated Hospital, Zhejiang University School of Medicine, Hangzhou 310009, China.; 2 Zhejiang University Cancer Institute, Key Laboratory of Cancer Prevention and Intervention, China National Ministry of Education, the Second Affiliated Hospital, Zhejiang University School of Medicine, Hangzhou 310009, China.; 3 Department of Hematological Malignancies Translational Science, Gehr Family Center for Leukemia Research, Hematologic Malignancies and Stem Cell Transplantation Institute, Beckman Research Institute, City of Hope Medical Center, Duarte, CA 91010.; 4 Institute of Genetics, Zhejiang University and Department of Genetics, Zhejiang University School of Medicine, Hangzhou 310058, China.; 5 National Clinical Research Center for Hematologic Diseases, the First Affiliated Hospital of Soochow University, Suzhou 215006, China.; 6 Zhejiang Provincial Key Laboratory for Cancer Molecular Cell Biology, Life Sciences Institute, Zhejiang University, Hangzhou 310058, China.

**Keywords:** B cell lymphoma, miR-21, NL101, c-Myc, Mxd1

## Abstract

**Background:** NL101 has shown activities against multiple myeloma and acute myeloid leukemia, but its anti-lymphoma activity remains unknown. The transcription factor c-Myc is frequently dysregulated in aggressive B cell lymphomas such as double-hit lymphoma, for which the standard of care is still lacking. A novel approach to target c-Myc needs to be explored. Although the role of oncogenic microRNA-21 (miR-21) was well established in an inducible mice model of B cell lymphoma, whether targeting miR-21 could inhibit the growth of B cell lymphoma and its underlying mechanisms is unclear.

**Methods:** We used MTT assay and flow cytometry to determine the inhibitory effect of NL101 on the cell proliferation of B cell lymphoma *in vitro*. The lymphoma xenograft mice models were generated to evaluate the anti-lymphoma function *in vivo*. Western blot and qPCR were applied to measure the expression levels of protein and microRNA, respectively. To investigate the mechanisms of action in NL101, we used genechip to profile differentially-expressed genes upon NL101 induction. Luciferase reporter system and chromatin immunoprecipitation were used for the validation of target gene or miRNA.

**Results:** Nl101 significantly inhibited B cell lymphoma proliferation through induction of cell cycle arrest and apoptosis. NL101 suppressed the growth of B cell lymphoma *in vivo* and prolonged the survival of lymphoma xenograft models. Gene expression profiling revealed that miR-21 was significantly decreased upon the induction of NL101 in B cell lymphoma. The miR-21 level was associated with the sensitivity of NL101. miR-21 inhibited Mxd1 expression via directly combining to Mxd1 3'-UTR; c-Myc activated miR-21 expression by directly binding to the miR-21 promoter.

**Conclusion:** NL101 significantly inhibited the growth of B cell lymphoma *in vitro* and *in vivo*. The novel c-Myc/miR-21/Mxd1 positive-feedback loop is critical for the maintenance of B cell lymphoma survival. Targeting miR-21 to block c-Myc/miR-21/Mxd1 loop represents a novel potential strategy of c-Myc-directed therapy.

## Introduction

MicroRNAs (miRNAs) are 21-23 nucleotide small non-coding RNAs that regulate gene expression at the posttranscriptional level, leading to target mRNA degradation or translational inhibition. The widespread dysregulation of miRNA expression is closely related to the pathogenesis of B cell lymphomas 1. MicroRNA-21 (miR-21), the most overexpressed miRNA in cancer, plays a critical role in various pathophysiological processes such as cell proliferation, differentiation, metabolism, and apoptosis 2, 3. Previous studies have shown that miR-21 is often upregulated in B cell lymphomas, and a high level of miR-21 confers chemoresistance and poor prognosis in the clinical setting 4, 5. Medina *et al*. demonstrated oncomiR addiction using a miR-21 conditional knock-in mice model that develops B cell lymphoma, thereby supporting miR-21 as a potential therapeutic target for cancer 6. The miR-21 inhibitors, alone or in combination with chemotherapeutic agents, have shown potent anti-cancer efficacies in mouse models of brain, breast, prostate, and pancreatic cancer 7, 8. Nevertheless, whether the targeting of miR-21 can inhibit the growth of B cell lymphoma remains unknown.

The transcription factor c-Myc is frequently dysregulated in hematological malignancies, especially in a large proportion of aggressive B cell lymphomas such as Burkitt lymphoma (BL) and diffuse large B cell lymphoma (DLBCL). The transgenic and knock-in mouse models in c-Myc driven by immunoglobulin heavy chain (IgH) locus enhancer highlights the critical role of c-Myc dysregulation in the development of B cell lymphoma 9, 10. The t(8;14) chromosomal translocation involving c-Myc rearrangement with IgH enhancer is considered as the cytogenetic hallmark of BL. Double-hit lymphomas (DHL), characterized by the rearrangement of c-Myc and Bcl-2 and/or Bcl-6, account for 5-15% of DLBCL; c-Myc also can be overexpressed without genetic rearrangements and is associated with concomitant Bcl-2 expression, resulting in a “double-expresser” phenotype 9, 11. c-Myc overexpression has been associated with inferior event-free survival in DLBCL 12. The median overall survival for patients with DHL is less than 24 months when treated with standard combination chemotherapy; even autologous or allogeneic hematopoietic transplantation does not improve the outcome of DHL 13. Despite the well-established role of c-Myc in aggressive B cell lymphoma, c-Myc-targeted therapy has yet to become clinically available for DHL and other c-Myc-driven B cell lymphomas.

c-Myc functions as a transcription factor that regulates the expression of a plethora of target genes and miRNAs, which promote or suppress the development of B cell lymphoma. To activate transcription, c-Myc heterodimerizes with Max to bind to a consensus sequence DNA element, enhancer box (E-box) of downstream target genes 14. Mxd1 competes with c-Myc for binding to Max to counteract c-Myc activity 15,16. Interestingly, c-Myc/Mxd1 has emerged as a critical regulatory axis in the growth of head and neck carcinoma 17. It is therefore likely that blocking c-Myc/Mxd1 axis serves as a novel strategy to target aberrant c-Myc function, which remains to be explored in B cell lymphomas.

In this study, we found that c-Myc, miR-21 and Mxd1 form a positive-feedback loop, which is pro-survival for B cell lymphoma; NL101, a DNA/HDAC dual-targeting small compound 18, 19, can effectively inhibit the growth of B cell lymphoma through blocking c-Myc/miR-21/Mxd1 loop.

## Materials and Methods

### Reagents

NL101 injection was supplied by Hangzhou Minsheng Institute of Pharmaceutical Research (Hangzhou, China). For mice model, NL101 injection was diluted with phosphate buffered solution (PBS) to 15 mg/mL.

### Cell lines

OCI-LY3 and Ramos were purchased from Cobioer Biotechnology Co., Ltd (Nanjing, China); OCI-LY10 was from Guandao Bioengineering Co., Ltd (Shanghai, China); U2932, Raji, Rec-1, Jeko-1, Maver-1 and JM1 were purchased from the American Type Culture Collection (ATCC). OCI-LY3 and Raji cells were cultured in IMDM (Gibco, US), HEK293T cells were cultured in DMEM (Gibco, US), the rest of cell lines were cultured in RPMI 1640 (Gibco, US). All mediums were supplemented with 10% fetal bovine serum (FBS) and 1% penicillin/streptomycin solution.

### Primary lymphoma cells

Peripheral blood, pleural fluid and ascites were obtained from patients with lymphoma, and mononuclear cells were isolated using Ficoll solution (Solarbio, China) and cultured in IMDM with 20% FBS. The inclusion criteria for the patients were: chronic lymphocytic leukemia or B cell lymphomas with pleural and/or abdominal dissemination, which were confirmed by pathology. All patients provided written informed consent. The study conformed to the Declaration of Helsinki, and was approved by the Institutional Review Board of the Second Affiliated Hospital, Zhejiang University School of Medicine.

### Animal models

NOD-SCID mice at the age of 4-6 weeks were purchased from Shanghai SLAC Laboratory Animal Co. Ltd, Shanghai, China, and housed at the Experimental Animal Research Center of Zhejiang Chinese Medical University. To establish the lymphoma xenograft model, 5×10^6^ Ramos cells were injected into the right axillary region, tumor size was measured daily, and tumor volume was calculated using the equation: Volume (mm^3^) = [maximal diameter (mm) × minimum diameter (mm^2^)]/2. The mice were randomly divided into vehicle- and NL101-treated groups, and 15 mice were initially included in each group. When the volume of xenograft tumor reached 100 mm^3^, NL101 was given 15 mg/kg intravenously every other day for 2 weeks. For the control group, an equivalent volume of PBS was given. Two weeks after treatment, 5 mice were euthanized to remove the tumors. The remaining mice were continued to be followed up. Mice were sacrificed when tumor sizes reached 2 cm^3^, and tumors were excised and weighed. The body weight was also measured every day. The overall survival rate was measured from the date of transplantation until death.

### MTT assay

Cell proliferation was determined by the MTT [3-(4,5-dimethylthiazol-2-yl)2 2,5-diphenyl tetrazolium bromide] assay kit (Sangon Biotech, China) according to the manufacturer's instructions. Cells were briefly seeded in 96-well plates and treated with gradient concentration of NL101 for 48 h. MTT was added at a final concentration of 0.5 mg/mL, and the cells were incubated for another 4 h. The supernatant was removed, DMSO was added, and the absorbance at 570 nm was read by a microplate reader.

### Cell cycle analysis

Cells seeded at 2
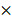
10^5^ per well were treated with NL101 of the indicated concentration for 48 hours, and then collected and fixed with 100% ethanol for 30 min. The fixed cells were centrifuged and re-suspended in 0.5 mL of phosphate-buffered saline solution containing 50 µg/mL each of RNase A and propidium iodide (PI, Invitrogen, US). The stained cells were analyzed in a fluorescence-activated cell sorter within 4 h. The percentages of cells in the G0/G1, S, and G2 -M phases were determined using the CellQuest program (BD Biosciences).

### Apoptosis assay

After NL101 treatment for 48 h, the cells were collected and stained with fluorescein isothiocyanate-annexin V and propidium iodide using AV-FITC/PI apoptosis detection kit (Keygen, China), according to the manufacturer's instructions. Annexin V and propidium iodide positive cells were determined by flow cytometry.

### Western blot analysis

Cells were lysed with RIPA lysis buffer containing protease inhibitors (Thermo Scientific, US) at 4 °C for at least 30 min. Protein concentration was measured using BCA protein assay kit (Thermo Scientific, US). Then, equal amount of proteins was separated by 8-12% SDS-PAGE, transferred to a polyvinylidene fluoride (PVDF) membrane (Milliopre, US), and probed with the indicated antibodies, followed by ECL detection. Antibodies against pATR, pCHK1, pATM, pCHK2, γH_2_AX and Parp1 were purchased from Santa Cruz Biotechnology, US; antibodies against Caspase 3, cleaved caspase 3 were from Cell Signaling Technology, US; anti-c-Myc and anti-Mxd1 were purchased from Abcam, US.

### Gene expression profiling

Cells were treated with 10 μM NL101 for 0, 3 and 6 h, and then subjected to total RNAs isolation using Trizol reagent (Invitrogen). Affymetrix Human Genome U133 plus 2.0 array, which contained 54,675 probesets, was used for genechip analysis performed in Shanghai Biotechnology Corporation, China.

### RNA isolation and quantitative polymerase chain reaction (qPCR)

miRNAs were extracted using the miRNA Kit (Omega, US) and reverse transcribed by TaqMan MicroRNA Reverse Transcription kit (Thermo, US). Total RNA was extracted by Trizol (Thermo, US) and reverse transcribed to cDNA. qPCR was performed using the SYBR Green PCR kit (Yeasen, China). miRNA and mRNA expressions were respectively normalized to U6 and GAPDH as internal controls. The primer sequences (Sangon, Shanghai, China) were as follows: miR-21, forward, 5'-GCAGTAGCTTATCAGACTGATG-3' and reverse, 5'-GGTCCAGTTTTTTTTTTTTTTTCAAC-3'; U6, forward, 5'-CTCGCTTCGGCAGCACA-3' and reverse, 5'-AACGCTTCACGAATTTGCGT-3'; GAPDH, forward, 5'-GGAGCGAGATCCCTCCAAAAT-3' and reverse, 5'-GGCTGTTGTCATACTTCTCATGG-3'. The relative expression level of each transcript was calculated by the 2^-ΔΔCt^ method. Each experiment was performed in triplicate.

### Lentiviral vector construction and infection

The c-Myc cDNAs were generated by RT-PCR using total RNAs purified from Ramos cells. The primers were as follows: forward, 5'-TGATCTAGAGCTAGCGAATTCGCCACCCTGGATTTTTTTCGGGTAGTG-3', and reverse 5'-GTAGTCAGCGGCCGCGGATCCTTACGCACAAGAGTTCCGTAG-3'. The cDNA was cloned into pCDH-EF1-MCS-(PGK-Puro) vector (SBI, US). The genomic DNA of Ramos was used as a template to amplify pri-miR-21 with the following primers: forward, 5'-AACGCGCGGTGACCCTCGAGTTCGATCTTAACA-3', and reverse 5'-TAAATAAAAAGCGGCCGCACAAAAGACTCTAAGTGCCAC-3'. The resulting pri-miR-21 fragment was inserted into pLL3.7 plasmid (Addgene, US). To generate a construct that expresses short harpin RNA (shRNA), shRNA duplexes for c-Myc (TRCN0000039640) were purchased from Sigma and inserted into pLKO.1 vector (Addgene, US). The lentiviral constructs and packaging vectors (pMD2.G and psPAX2) were co-transfected into HEK293T cells with LipoFliter (Hanbio, China). The packaged recombinant lentiviruses were harvested from the supernatant at 48h post-transfection and concentrated by ultrafiltration (Millipore, US). Ramos and OCI-LY10 were infected with lentivirus at a mutiplicity of infection (MOI) of 50 using 10 μg/mL polybrene (Sigma, US).

### Transfection of antago-miR-21

Ramos and OCI-LY10 cells were seeded in 24-well plates and transfected with antago-miR-21 or antago control (all 100 nM) (Ribiobio, Guangzhou, China) according to the manufacturer's instructions. The efficiency of the transfection was determined by qPCR.

### Generation of luciferase reporter constructs

The 3'-UTRs containing predicted miR-21 binding sites were amplified by PCR using the following primers: Mxd1-3'UTR forward, 5'-CAGTAATTCTAGGCGATCGTTTGAAGCATCCAAGAATTC-3', and reverse 5'-TTTTATTGCGGCCAGCGGCCTCAAGACCTCAAAAATATGAATG-3'; PDCD4-3'UTR forward, 5'-AATTCTAGGCGATCGCTCGAGTTTTTTTTTGTTTTTCGAGGGGG-3', and reverse 5'-ATTTTATTGCGGCCAGCGGCCGCCCAGTTTTTGTAGAAAAAT-3'. The 3'UTRs were then cloned into a psiCHECK2 vector (Promega, US). The mutant Mxd1 3'UTRs at miR-21 binding sites were generated using Multi Site-Directed Mutagenesis Kit (Yeason, Shanghai, China), the wild type psiCHECK2-Mxd1-WT was used as a template, and the primers were as follow: psiCHECK2-Mxd1-MUT1, forward 5'-CCAAAGCATGAGTTGAAAGCAGCATTTAAGAGCACTTAA-3', and reverse 5'-TGCTTTCAACTCATGCTTTGGAGTTCTGAGATCAGGA-3'; psiCHECK2-Mxd1-MUT2, forward, 5'-CGACTTTGTTGCCGTTTCAGTCATGACAAAACAGAGCACATGTATATGTA-3', and reverse, 5'-TCTGTTTTCTCATGAGTCAAAGCGCAACAAAGTCGGAGACCACTTAAGGG-3'. To generate psiCHECK2-Mxd1-MUT3, psiCHECK2-Mxd1-MUT 1 was used as a template, and site-directed mutagenesis was done using mutant 2 primers.

The miR-21 core promoter (-1000 to +1) was amplified by PCR using forward primer 5'-GGTACCGAGCTCTTACGCGTGCTTTTTAGGCTTGTCACAA-3' and reverse primer 5'-GATCGCAGATCTCGAGAGGTGGTACAGCCATGGAGA-3' and cloned into a pGL3.0 luciferase vector (Promega, US), and the resulting construct was designated as pGL3.0-miR-21-WT. The mutant constructs were generated using Multi Site-Directed Mutagenesis Kit with the following primers: pGL3.0-miR-21-MUT1, forward, 5'-GTGTCTTACTACTCACCTGACACCATTTTTTA-3', and reverse 5'-GATCTCTAGTTAAGACACCAACCAGATTTTCCTT-3'; pGL3.0-miR-21-MUT2, forward, 5'-TCTGGTCGAATAAGCTAAAAGTAAAAAATCAAAACGC-3', and reverse, 5'-CCGTTCTGTTACTCTATTATTCTTAGTGTGATTTTT-3'; pGL3.0-miR-21-MUT3, forward, 5'-CCGTTCTGTTACTCTATTATTCTTAGTGTGATTTTT-3', and reverse, 5'-ATAGAGTAACAGAACGGCAAGAAAACTGGGTTA-3'. All constructs were confirmed by sequencing.

### Luciferase assay

For the target sites of miR-21 on Mxd1 3'-UTR, 200 ng of miR-21 mimic, 500 ng wild-type or mutant psiCHECK2-Mxd1 was co-transfected into HEK293T using LipoFiter. PDCD4 3'UTR was used as a positive control. For the miR-21 promoter assay, 500 ng pCDH-c-Myc, 150 ng wild type or mutated pGL3.0-miR-21, was co-transfected into HEK293T using LipoFiter. pGL3.0 and pCDH mock vectors were transfected as controls for luciferase assay and transfection, respectively. miR-21 mimic and mimic control were purchased from Ribobio (Guangzhou, China). Forty eight hours after HEK293T transfection, a luciferase reporter assay was performed using Dual-Luciferase Reporter Assay System (Promega, US) according to the manufacturer's protocol.

### Chromatin immunoprecipitation assay

With the EZ-Magna ChIP A/G Assay Kit (Millipore, US), chromatin immunoprecipitation assays were performed following the manufacturer's protocol. The antibody against c-Myc was purchased from Abcam (US). Primers flanking the c-Myc binding sites on the miR-21 promoter were used for qPCR, and the sequences were as follows: forward, 5'-CATTGCACACCCTCTGGGGAAATT-3, and reverse 5'-CAAGACTTCATTCTAAATACACAG-3'.

### Bioinformatic analysis

The TargetScan (http://www.targetscan.org/vert_71/) and miRbase (http://www.mirbase.org/) databases were searched to identify the potential miR-21 target genes and their binding sites. The predicted target genes and differentially-expressed genes derived from the genechip analysis were imported into the online Draw Venn Diagram tool (http://bioinformatics.psb.ugent.be/webtools/Venn/) to identify the overlapping genes. The single cell sequencing dataset, CancerSEA (http://biocc.hrbmu.edu.cn/CancerSEA/), was used to evaluate functions of miR-21 target genes. miR-21 promoter sequence was extracted from the USCS database (http://genome-asia.ucsc.edu/cgi-bin/hgGateway?redirect=manual&source=genome.ucsc.edu). A LASAGNA-Search 2.0 dataset (https://biogrid-lasagna.engr.uconn.edu/lasagna_search/) was obtained to predicate the binding sites of c-Myc on miR-21 promoter.

### Statistical analysis

Statistical analyses were carried out with GraphPad Prism (GraphPad Software). The comparisons of the expression levels, luciferase activities, tumor volumes and outcome among different groups was evaluated by one-way analysis of variance (ANOVA) or the Student's test. The Spearman's rank correlation coefficient was used as a statistical measure of association and |R| ≥ 0.3 was considered significantly correlated. All the statistical tests were 2-sided, and a value of *P* ≤ 0.05 was considered statistically significant.

## Results

### NL101 inhibits B cell lymphoma growth *in vitro* and *in vivo*

**Figure 1:**
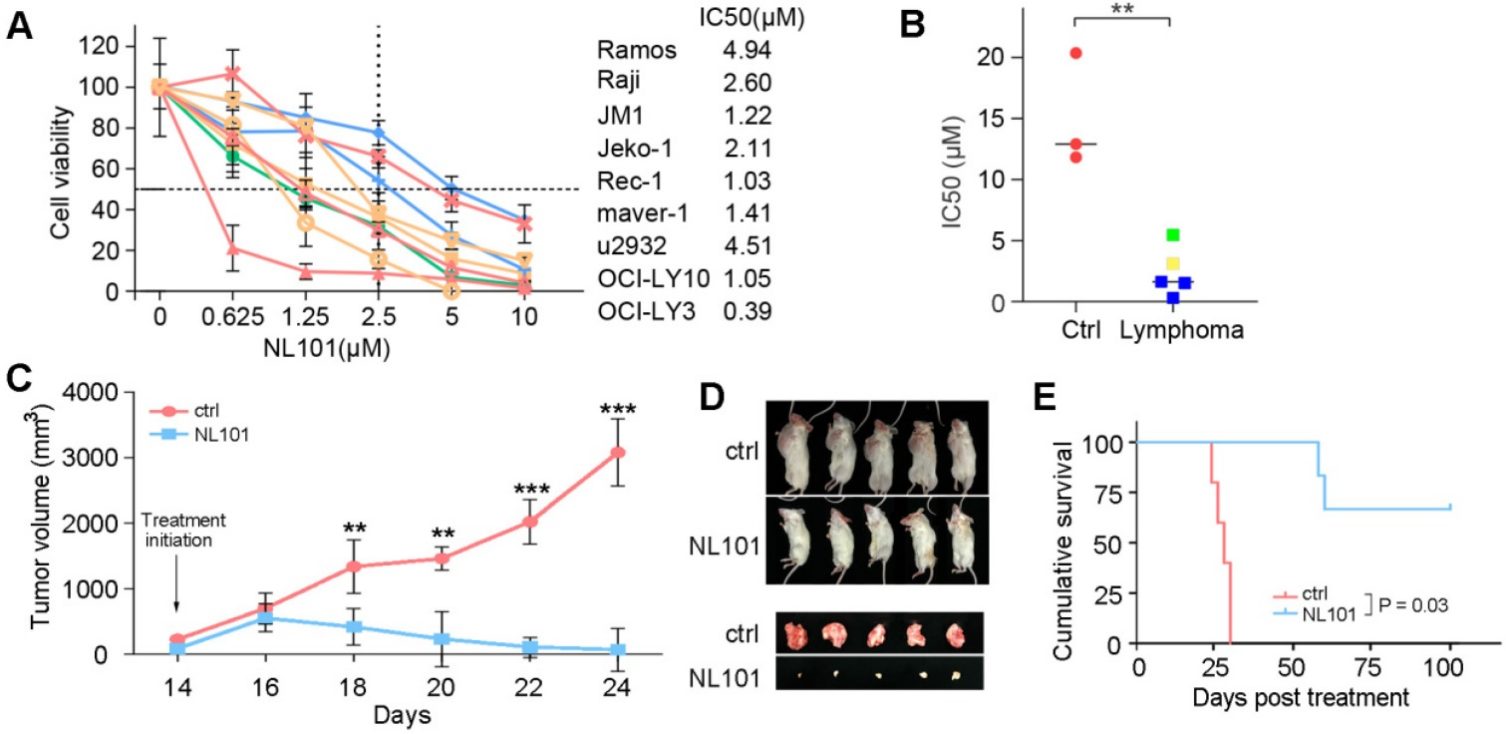
NL101 inhibits the growth of B cell lymphoma *in vitro* and *in vivo*. **A.** Nine B cell lymphoma cell lines were incubated with indicated concentrations of NL101 for 48 h, and the viability was analyzed by MTT assay. **B.** IC50 of NL101 on primary cells from 3 healthy volunteers (left; red dots) and 5 patients with B cell lymphoma (right; green square represents the sample from the pleural fluid of a DLBCL patient; yellow square represents the sample extracted from the ascites of a DLBCL patient; blue squares are samples from 3 CLL patients' peripheral blood). **C.** Mice bearing subcutaneous Ramos xenograft were randomized to receive PBS (control) and NL101 (15 mg/kg, iv, every other day for 2 weeks), respectively, tumor volumes were calculated every other day. *, P < 0.05; **, P < 0.01; ***, P < 0.001. **D.** Images of mice and corresponding tumors of control and NL101 groups. **E.** Survival of mice xenograft model analyzed with a Kaplan-Meier curve.

To determine the effect of NL101 on cell proliferation, we tested NL101 in 9 B cell lymphoma cell lines with MTT assay. As shown in Figure. 1A, all cell lines showed reduced cellular viability after NL101 treatment, and the IC50s at 48 h ranged from 0.625-4.994 μM. We also determined the cytotoxicity of NL101 against primary cells. Compared with the normal cells from healthy volunteers, for which median IC50 were 12 μM, the primary B cell lymphoma cells were more susceptible to NL101-induced cell death, with a median IC50 of 3.5 μM (Figure 1B). Next, we established the xenograft mouse model and evaluated the anti-lymphoma activity of NL101 *in vivo*. NOD-SCID mice were injected with Ramos cells subcutaneously and were subject to NL101 or vehicle treatment once the lymphoma xenografts developed. As a result, NL101 significantly inhibited lymphoma growth starting from day 4 after the first dose of NL101, P < 0.05 (Figure 1C), and the excised tumors are shown in Figure 1D. Moreover, NL101-treated mice showed a prolonged survival rate as compared to vehicle-treated mice; P < 0.05, and > 50% of mice in the NL101 group had been alive for more than 100 days while all the mice in the control group died before day 50 (Figure 1E). There was no significant difference between body weights of vehicle- and NL101-treated mice (data not shown). Taken together, these data suggest that NL101 inhibits the growth of B cell lymphoma *in vitro* and *in vivo*.

### NL101 induces cell cycle arrest and apoptosis through DNA damage response

**Figure 2:**
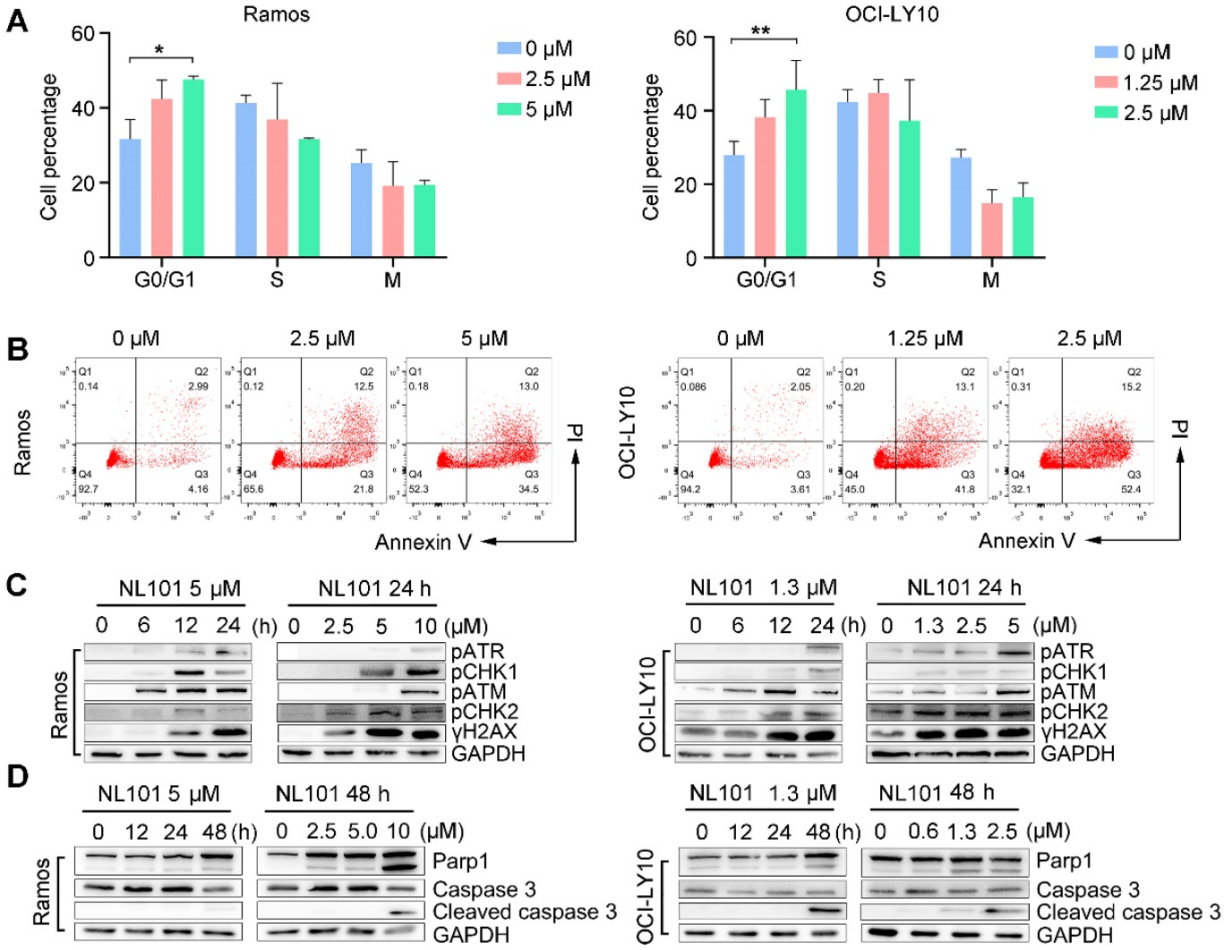
NL101 induces cell cycle arrest and apoptosis.** A.** Ramos and OCI-LY10 cells were treated with NL101 at indicated concentrations for 48h and then subjected to FACS analysis. **B.** Annexin-V labeling of Ramos and OCI-LY10 cells after treatment with indicated doses of NL101 for 48h and evaluated by flow cytometry. **C.** Dose- and time-responses of NL101 in Ramos and OCI-LY10 cells, cell lysates were used in western blot to detect proteins implicated in the DNA damage response, and GAPDH was determined as a loading control. **D.** Dose- and time-response changes of proteins involved in apoptosis after NL101 treatment of Ramos and OCI-LY10 cells. *, P < 0.05; **, P < 0.01.

To determine whether NL101-induced growth inhibition is due to cell cycle arrest and/or apoptosis, we performed FACS analysis on Ramos and OCI-Ly10 cells with and without NL101 treatment. When NL101 was given for 24 h, the percentage of G0/G1 fraction increased from 32% to 47% in Ramos cells, and from 27% to 45% in OCI-Ly10 cells (Figure 2A); When NL101 was given for 48h, the annexin V/PI staining assay showed that the percentage of apoptotic cells increased in a dose-dependent manner (Figure 2B). Since NL101 is designed as a bendamustine derivative, we questioned if cell cycle arrest and apoptosis were caused by DNA damage. Phosphorylation of ATM, ATR, CHK1, and CHK2 are common events in DNA damage response. As shown in Figure 2C, when Ramos and OCI-Ly10 cells were treated with NL101, ATM and CHK2 were phosphorylated earlier than ATR and CHK1 in a dose- and time-dependent manner. When activated, ATM phosphorylates γH2AX at the end of the double-strand breaks as phosphorylated γH2AX also increased following NL101 treatment (Figure 2C). As apoptotic markers, cleaved forms of PARP and caspase 3 were detectable in both Ramos and OCI-LY10 cells upon NL101 treatment (Figure 2D). Thus, these data suggest that NL101 induces cell cycle arrest and apoptosis through DNA damage response in B cell lymphomas.

### Downregulation of miR-21 contributes to NL101-induced B cell lymphoma growth inhibition

**Figure 3:**
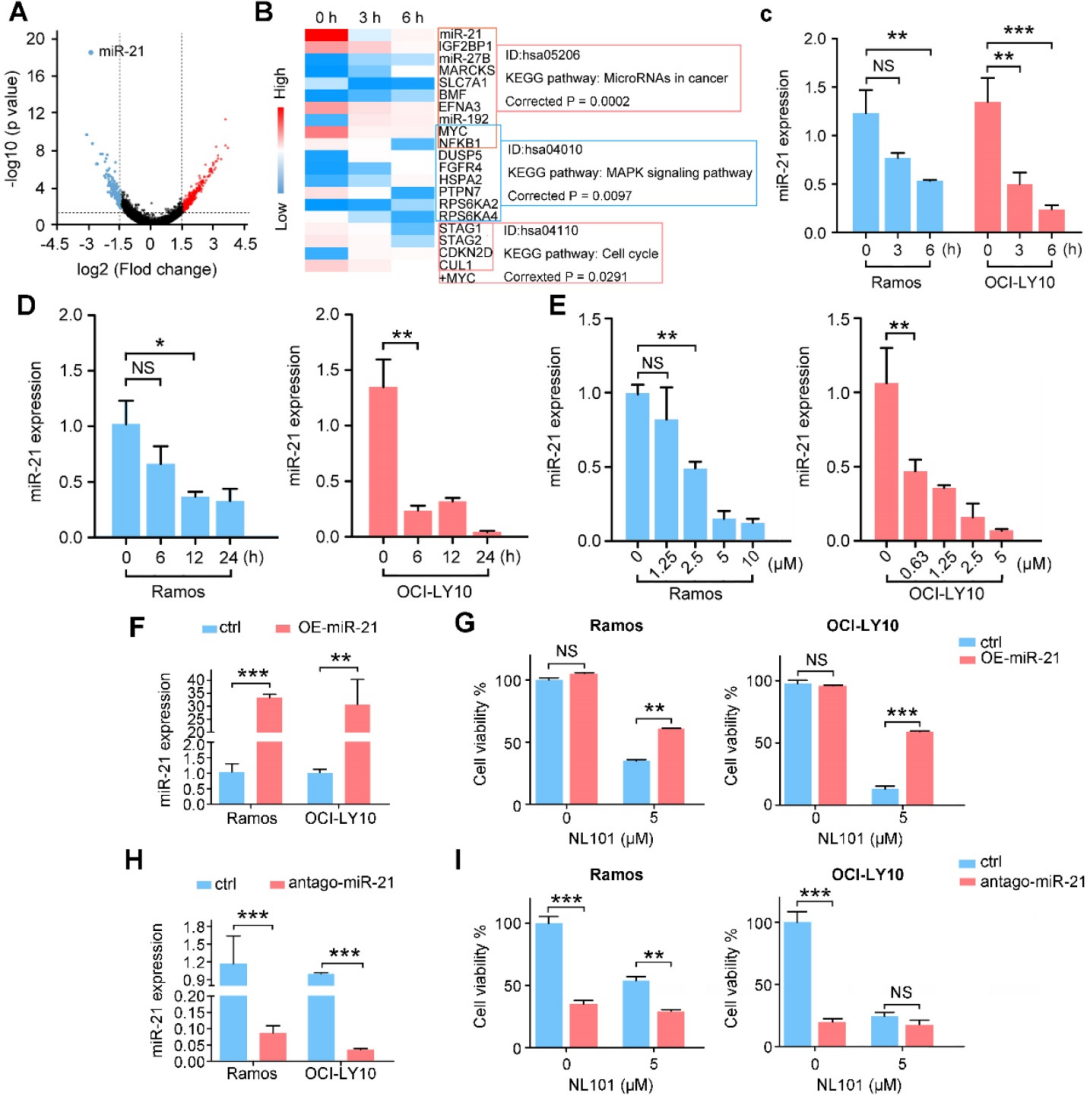
NL101 inhibits miR-21 expression.** A.** The volcano plot was used for enabling visualization of the relationship between fold change (FC) and statistical significance. The vertical lines correspond to 1.5-log FC in up and down expression, while the horizontal line represents a *p-*value of 0.05. Therefore, the black point in the plot represents genes with no statistical differences (-1.5 < log FC < 1.5, P > 0.05), the red point represents upregulated genes (log FC > 1.5, P < 0.05) and the blue point represents downregulated genes (log FC < -1.5, P < 0.05) with statistical significance. **B.** Heatmap analysis showed the hierarchical clustering of differentially-expressed genes in OCI-LY10 cells receiving 10 µM NL101 for 0, 3, 6 h. **C.** Ramos and OCI-LY10 cells were treated with 10 µM NL101 for 0, 3, 6 h, miR-21 level was determined by qPCR. **D.** Ramos and OCI-LY10 cells were treated with 5 µM NL101 for 0, 6, 12 and 24 h, miR-21 level was determined by qPCR.** E.** Ramos and OCI-LY10 cells were treated with NL101 at indicated concentration for 12 h, miR-21 level was determined by qPCR. **F.** Ramos and OCI-LY10 were transfected with 10 nM pLL3.7-control-miR or pLL3.7-miR-21. Two days after transfection, miR-21 level was determined by qPCR. **G.** Ramos and OCI-LY10 were transfected with 10 nM pLL3.7-control-miR or pLL3.7-miR-21 for 2 days, then untreated or treated with 5 µM NL101 for 24 h, and cells were counted.** H.** Ramos and OCI-LY10 were transfected with 100 nM antago-control miR or antago-miR-21. Two days after transfection, miR-21 level was determined by qPCR. **I.** Ramos and OCI-LY10 were transfected with 10 nM control antago-miR or antago-miR-21 for 2 days, and then untreated or treated with 5 µM NL101 for 24 h, cells were counted. *, P < 0.05; **, P < 0.01; ***, P < 0.001; ctrl, control group.

To investigate the underlying mechanisms by which NL101 suppresses the growth of B cell lymphoma, we performed gene expression profiling to identify critical genes responsible for the sensitivity of NL101. Total RNAs were purified from OCI-LY10 cells treated with NL101 for 0, 3 and 6 h, and used to hybridize the Affymetrix U133-plus 2 GeneChip. A total of 553 genes (with fold change 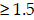) were differentially expressed, including 361 upregulated and 192 downregulated genes, and miR-21 was the most downregulated gene with more than 1000-fold change, P = 2.73E-19 (Figure 3A). The top 20 differentially-expressed genes induced by NL101were classified into 3 major KEGG pathways: microRNA, MAPK signaling and cell cycle regulation (Figure 3B). Although the role of miR-21 had been well recognized in B cell lymphoma model, whether targeting miR-21 could suppress B cell lymphoma remains unclear. Therefore, we sought to characterize the role of miR-21 in NL101-induced growth inhibition of B cell lymphoma.

We first validated the microarray data using qPCR in OCI-LY10 and Ramos cells. As shown in Figure 3C a reduction in miR-21 expression was noted as early as 3 h after NL101 treatment, suggesting that miR-21 downregulation is likely a primary but not secondary effect of NL101. The time- and dose-response experiments showed that NL101 significantly inhibits miR-21 expression in both lymphoma cell lines (Figure 3D-E). To determine the effect of miR-21 expression on cell viability following NL101 treatment, miR-21 levels were altered in Ramos and OCI-LY10 cells. We found that, miR-21-overexpressed cells were more resistant to NL101-induced cell death than parental cells (Figure 3F-G). Moreover, antago-miR-21, a chemically engineered miR-21 inhibitor capable of knocking down endogenous miR-21, induces significant cell death, suggesting that miR-21 is a critical pro-survival factor; antago-miR-21 also sensitized cells to NL101-induced cell death, as more cells died when treated with both agents than those received NL101 alone (Figure 3H-I). Taken together, these data suggest that miR-21 is a critical determinant of NL101 sensitivity in B cell lymphoma.

### Mxd1 is a novel target of miR-21

**Figure 4:**
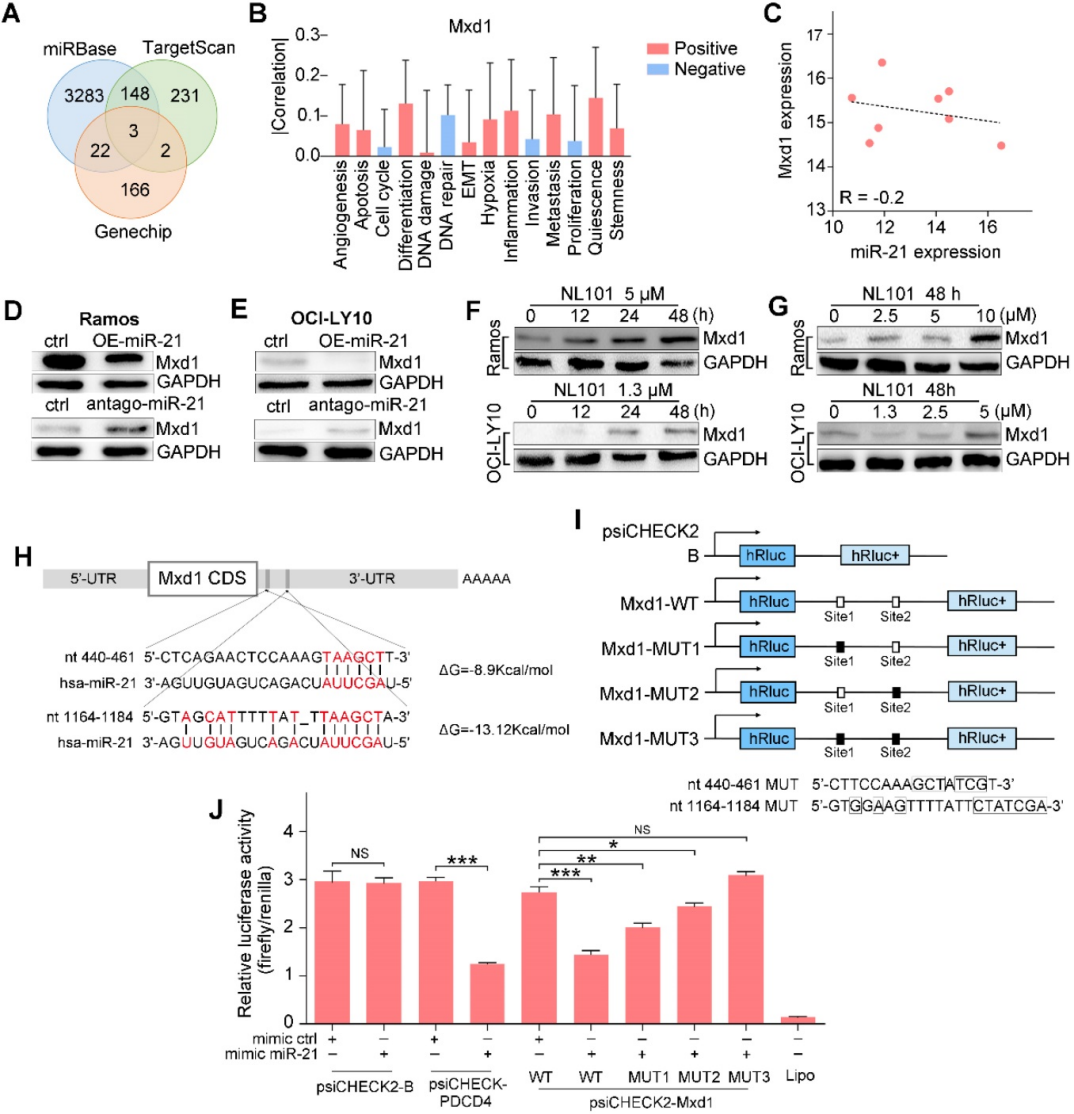
miR-21 targets the 3'UTR of the Mxd1 and downregulates its expression.** A.** Venn map of potential miR-21 target genes in TargetScan, miRbase and Genechip. **B.** The predicted functions of Mxd1 by CancerSEA database.** C.** Analysis of the correlation of Mxd1 mRNA and miR-21 levels in human B cell lymphoma by the Spearman rank correlation coefficient.** D, E** Ramos (D) and OCI-LY10 (E) were transfected with 50 nM miR-21 mimics or antago-miR-21 or controls. Two days after transfection, cell extracts were prepared and the levels of Mxd1 and control GAPDH were determined by Western blot.** F.** Ramos and OCI-LY10 cells were treated with indicated concentration of NL101 for 0, 12, 24 and 48 h, cell extracts were prepared and the levels of Mxd1 and control GAPDH were determined by Western blot. **G**. Ramos and OCI-LY10 cells were treated with indicated concentration of NL101 for 48 h, cell extracts were prepared and the levels of Mxd1 and control GAPDH were determined by Western blot. **H.** Schematic illustration of the miR-21 binding sites at the 3'UTR of Mxd1 gene.** I.** schematic presentation of the psiCHECK luciferase reporter constructs containing wild-type or mutant miR-21 binding sites, where the nucleotide substitutions at the critical nucleotides are shown in boxes. **J.** HEK293T cells was co-transfected with miR-21 mimics or control, and the psiCHECK luciferase reporter constructs containing Mxd1 wild-type or mutant sites. A dual-luciferase reporter assay was performed. *, P < 0.05; **, P < 0.01; ***, P < 0.005; NS, no significance; ctrl, control group.

To gain insight into the mechanism underlying the regulatory role of miR-21 in B cell lymphoma survival, we searched TargetScan and miRbase databases and identified a number of potential miR-21 targets. Since miR-21 inhibits downstream transcription, we overlapped the database hits with the genechip data, as shown in the Venn diagram, Mxd1, MAP2K3 and PAIP2B were identified (Figure 4A). CancerSEA, a cancer single-cell functional state atlas, revealed that Mxd1 was closely related to the regulation of apoptosis and DNA repair (Figure 4B). Mxd1 functions as a c-Myc antagonist, and given the essential role that c-Myc plays in B cell lymphomagenesis, we wanted to know whether Mxd1 could be a novel miR-21 target. According to our analysis of the database, an inverse correlation was observed between miR-21 and Mxd1 expressions, R = -0.2 (Figure 4C). Western blot showed that the overexpression of miR-21 reduced Mxd1 expression (Figure 4D), whereas antago-miR-21 significantly enhanced Mxd1 expression in Ramos and OCI-LY10 cells (Figure 4E). Upon NL101 induction, in parallel with miR-21 decreased expression (Figure 3D-E), Mxd1 increased in time- and dose-dependent manner in Ramos and OCI-LY10 cells (Figure 4F-G). We next asked whether miR-21 directly targets Mxd1 3'UTR, a general mechanism by which miRNAs regulate target expression. Using TargetScan and miRbase, we identified two putative miR-21 binding sequences, i.e., nt 440-461 and nt 1164-1184, located in the 3'-UTR of Mxd1 gene (Figure 4H). To test whether these binding sites are functional, we cloned the Mxd1 3'UTR fragment of nt 241-1384, which was designated as Mxd1-WT, in front of a luciferase reporter within psiCHECK2 plasmid; We also generated mutations at miR-21 binding sites and cloned the mutant 3'UTRs, i.e., Mxd1-MUT1, Mxd1-MUT2 and Mxd1-MUT3, into the same luciferase reporter, respectively (Figure 4G). Either wild-type or mutant Mxd1 3'UTR-luciferase reporter was co-transfected with miR-21 mimics in HEK293T cells. As shown in Figure 4H, miR-21 did not affect the luciferase activity of psiCHECK2 alone, but significantly inhibited the luciferase activity of psiCHECK2 containing PDCD4 3'-UTR, which serves as a positive control. Compared with the control mimic, miR-21 mimic significantly decreased the luciferase activity of Mxd1-WT (Figure 4H). Among Mxd1 3'UTR mutants, MUT1 are more responsive to miR-21 mimic than MUT2, and MUT3 completely abrogated the diminished effect by miR-21 mimic (Figure 4H), which indicate that both miR-21 binding sites are functional and the sequence nt 1164-1184 is the major binding site on Mxd1 3'-UTR. Together, these data suggest that Mxd1 is a novel direct target of miR-21.

### c-Myc activates miR-21 expression

**Figure 5:**
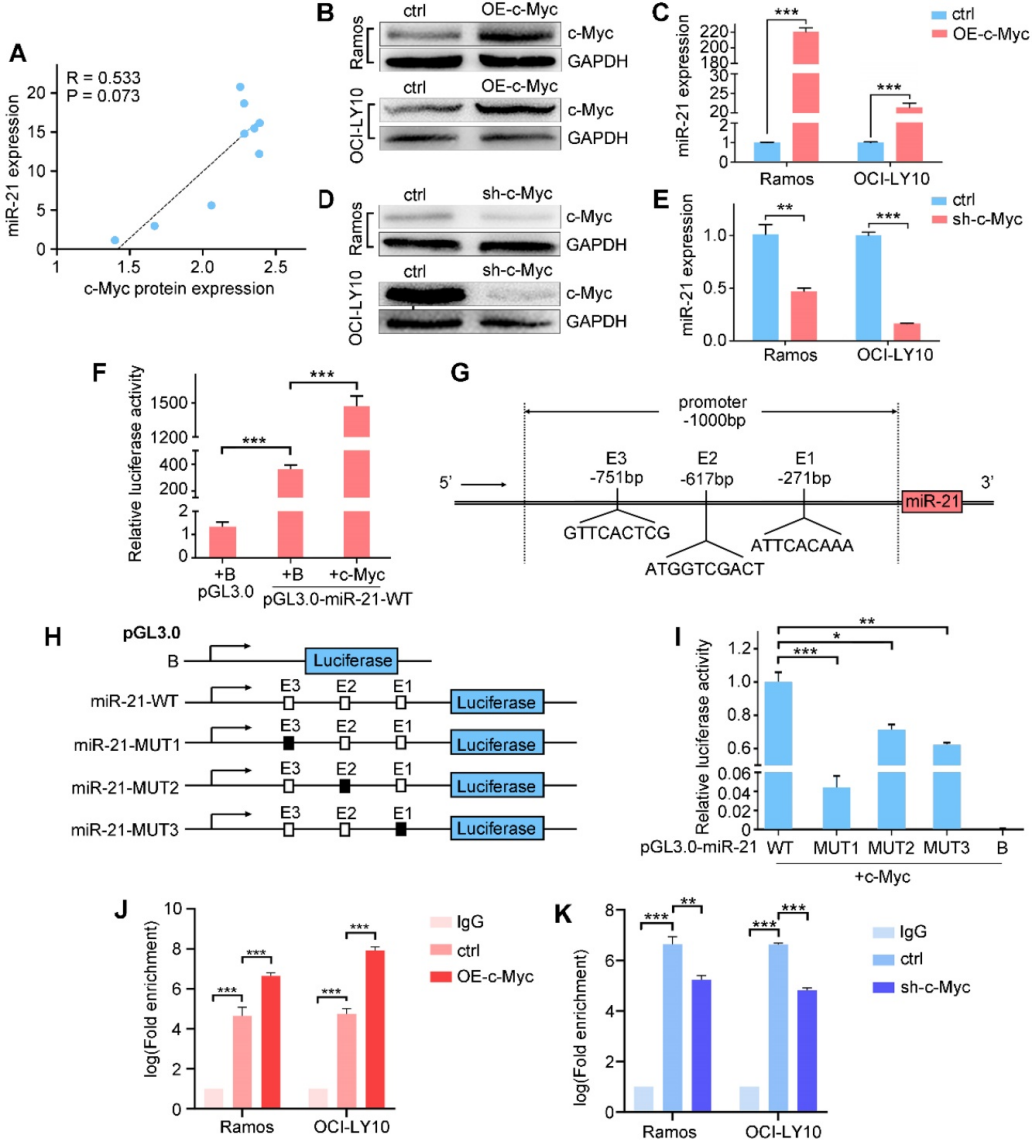
c-Myc transactivates miR-21 expression by binding to the pri-miR-21 promoter. **A.** Correlation between c-Myc protein and miR-21 expression in B cell lymphoma lines. **B, C.** Ramos and OCI-LY10 were transfected with pCDH vector or pCDH-c-Myc for 48h, cell extracts were prepared and the levels of c-Myc and control GAPDH were determined by Western blot (B), and the miR-21 level was determined by qPCR (C). **D, E.** Ramos and OCI-LY10 were transfected with pLKO vector (control) or pLKO-c-Myc for 48 h, cell extracts were prepared and the levels of c-Myc and control GAPDH were determined by Western blot (D), and the miR-21 level was determined by qPCR (E). **F.** HEK293T cells was transfected with control pGL3.0, pGL3.0-miR-21-WT, and pCDH-c-Myc, or both, 48 h after transfection, the cells were lysed, and luciferase activity was measured. **G.** Schematic diagram of the locations of c-Myc-binding sites at the miR-21 promoter region (-1000 bp to +1 bp). **H,** schematic presentation of the pGL3.0 luciferase reporter constructs containing wild-type or mutant E boxes. **I.** HEK-293T cells were transfected with the pGL3.0 wild-type or mutants with pCDH-c-Myc, the cells were lysed, and luciferase activity was measured. **J, K.** Transfections were performed as in B or D, cell extracts were prepared and ChIP assays were performed. *, P < 0.05; **, P < 0.01; ***, P < 0.001.

miR-21 directly targets Mxd1, which competes with c-Myc for binding to Max, thereby suppressing c-Myc-mediated transcriptional activation. We analyzed the expression database and found a positive correlation between miR-21 and c-Myc (Figure 5A); thus, we hypothesized that miR-21 transcription is regulated by c-Myc in a feedback loop in B cell lymphoma. To address this, we examined miR-21 and c-Myc expressions by qPCR and western blot, respectively, and found that the ectopic expression of c-Myc significantly induced miR-21 expression (Figure 5B-C), while knocking down c-Myc inhibited miR-21 expression (Figure 5D-E).

We next asked whether miR-21 is a direct transcriptional target of c-Myc. A 1000-bp promoter region was amplified and cloned into a pGL3.0 luciferase vector, of which the resulting construct was designated as pGL3.0-miR-21-WT. Compared with pGL3.0 alone, the luciferase activity of pGL3.0-miR-21-WT was significantly increased, and was further enhanced by c-Myc co-transfection (Figure 5F), indicating that c-Myc binding sites occur in the pri-miR-21 promoter. Using LASAGNA-Search 2.0 web tool, we found three putative consensus E box sequences in the pri-miR-21 promoter, i.e., GTTCACTCG, ATGGTCGACT and ATTCACAAA, respectively (Figure 5G). We generated pGL3.0 luciferase constructs containing mutant c-Myc-binding sites (Figure 5H). The wild-type or mutant reporter construct was co-transfected with c-Myc. As shown in Figure 5I, pGL3.0-miR-21-WT showed the highest luciferase activity, while the pGL3.0-miR-21-MUT1, -MUT2 and -MUT3 show diminished luciferase activity, suggesting that these binding sites are responsible for the c-Myc transcriptional activation of miR-21. Finally, chromatin immunoprecipitation showed that c-Myc overexpression enhanced, while c-Myc knockdown suppressed, its binding to the GTTCACTCG site in miR-21 promoter. Taken together, these data suggest that miR-21 are direct target of c-Myc (Figure 5J-K).

### NL101 targets miR-21 to block c-Myc/miR-21/Mxd1 loop

**Figure 6:**
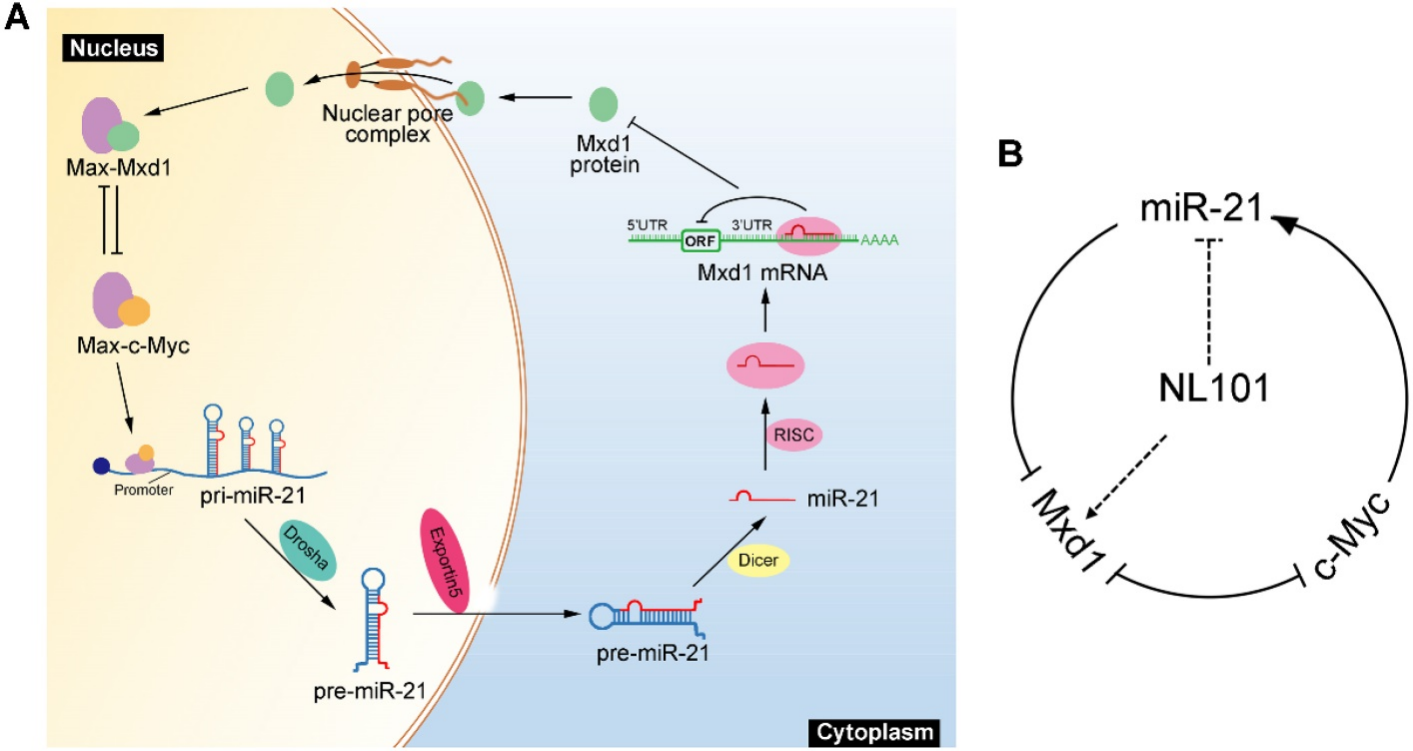
Schematic illustration of a positive feedback loop for the role of c-Myc/miR-21/Mxd1 in B cell lymphoma. **A**. c-Myc activation or overexpression enhances miR-21 expression, miR-21 in turn targets Mxd1 mRNA and down-regulates Mxd1 protein; and finally decreased Mxd1 promotes formation of c-Myc-Max heterodimer, leading to sustained c-Myc activation. **B**. Following NL101 treatment, the expression of miR-21 is decreased and Mxd1 is up-regulated, resulting in inhibition of c-Myc activity and further decreased expression of miR-21; thus, by targeting miR-21 to block c-Myc/miR-21/Mxd1 loop, NL101 can efficiently inhibit the growth of B cell lymphoma.

We showed that miR-21 is a pro-survival factor in B cell lymphoma. Mechanistically, miR-21 directly downregulates the tumor suppressor Mxd1, and diminished Mxd1 expression leads to c-Myc activation, which in turn induces miR-21 expression. Thus, c-Myc, miR-21 and Mxd1 form a positive-feedback loop, which represents a novel mechanism by which miR-21 promotes B cell lymphomagenesis (Figure 6A). A schematic representation of working model for NL101 is illustrated in Figure 6B. Through the significant inhibition of miR-21 expression, NL101 efficiently blocks the c-Myc/miR-21/Mxd1 positive feedback loop and inhibits the growth of B cell lymphoma.

## Discussion

Previous studies have demonstrated that NL101, also known as CY190602 or EDO-S101 18, 19, exhibits anti-cancer activities in acute myeloid leukemia and multiple myeloma 20-23, but little is known about its efficacy in lymphomas. In this study, we report that NL101 is highly active against B cell lymphomas such as DLBCL and BL, and NL101 significantly inhibits the expression of miR-21, which promotes lymphoma cell survival through modulating c-Myc/Mxd1 axis.

NL101 is originally designed as a bendamustine derivative, in which the side chain of bendamustine was replaced with the hydroxamic acid of suberoylanilide hydroxamic acid (SAHA); thus, there is DNA/histone deacetylase (HDAC) dual-targeting activity in NL101 18, 19. López-Iglesias *et al.* found that NL101 increases the acetylation of histones H3 and H4, and specifically inhibits the double strand break repair by the homologous recombination pathway 22. NL101 also inhibits HDAC to downregulate several DNA repair genes, including Tip60, CBP, MORF, and MSL1, resulting in DNA damage 19. In line with previous findings, we found that NL101 induces DNA damage in B cell lymphoma as ATR, ATM, CHK1 and CHK2 phosphorylation increase. Thus, NL101 enhances the cytotoxicity of DNA damage through the inhibition of HDAC-mediated DNA repair.

To characterize other NL101 functions besides DNA damage and HADC inhibition, we conducted an analysis of gene expression profiles following NL101 exposure and found that microRNA, MAPK signaling and cell cycle pathways are mostly affected. The essential role of microRNAs has been extensively studied in B cell lymphomas, subtypes of which share common and distinct miRNA signatures with diagnostic and prognostic implications 1. miR-21 is ubiquitously overexpressed in B cell lymphomas, and a high expression of miR-21 is associated with poor prognosis for patients with DLBCL. miR-21 addiction has been well-documented in a conditional B cell lymphoma model 6. Therefore, we sought to elucidate the mechanism by which NL101 targets miR-21 to suppress growth of B cell lymphoma. A few miR-21 target genes have been identified, but their roles in miR-21 mediated pro-survival in B cell lymphoma remain elusive. miR-21 activates the PI3K/AKT signaling pathway by directly suppressing FOXO1 and PTEN expressions in diffuse large B-cell lymphoma 24, 25. PDCD4 binds to eIF4A and selectively inhibits protein translation in the immune system. PDCD4 knockout mice develop spontaneous B-cell lymphomas 26, 27. Recently, Sahraei M *et al.* analyzed the function of miR-21 in noncancer cells of the tumor microenvironment and found that miR-21 expression in tumor associated macrophages (TAMs) is responsible for promoting tumor growth, and miR-21 inhibition in TAMs may improve cytotoxic T cell activity and reduce angiogenesis, leading to tumor suppression 28. We identified Mxd1 as a novel target of miR-21. First, Mxd1 expression correlates inversely with miR-21 levels, and NL101-induced miR-21 downregulation was accompanied by an increased Mxd1 expression; Second, Mxd1 3'UTR luciferase construct containing predicted miR-21 binding sites is specifically responsive to miR-21 mimic or antago-miR-21 treatment. Finally, mutations of the miR-21 binding site diminish the luciferase response to miR-21.

Mxd1 serves as a transcription repressor that antagonizes the transcriptional activation of c-Myc. Dual targeting c-Myc/Mxd1 axis has become an important regulatory mechanism in cancer pathogenesis. Salehi-Tabar *et al.* found that in head and neck squamous cell carcinoma, vitamin D receptor (VDR) suppresses c-Myc but enhances Mxd1 expression, and such opposing effect on c-Myc/Mxd1 axis leads to a significant transcriptional inhibition of c-Myc target genes 17. Based on bioinformatic analysis, miR-21 possibly does not target c-Myc due to the lack of miR-21 binding sites in c-Myc 3'UTR; on the contrary, there are 4 consensus E box sequences in the pri-miR-21 promoter. c-Myc induces miR-21 expression, enhances the luciferase activity of miR-21 promoter, and directly binds to E boxes of pri-miR-21. Taken together, we revealed c-Myc/miR-21/Mxd1 as a novel positive-feedback loop that plays a critical role in the maintenance of B cell lymphoma.

The human miR-21 gene is mapped to chromosome 17q23.2. The genomic locus encoding miR-21 is not amplified in most cancers. The regulation of miR-21 is a complex process that involves transcriptional and posttranscriptional steps along the biogenesis pathway 3. For example, STAT3-dependent miR-21 transcription was observed in IL-6 stimulated myeloma cells 29. Since miR-21 is a novel transcriptional target of c-Myc, frequent c-Myc overexpression may contribute to miR-21 upregulation in B cell lymphoma. Previously, c-Myc was shown to regulate other miRNAs, including let-7, miR-34b, miR-98, miR-150, miR-331, and miR-363 30,31. The miRNA cluster miR-17-92 is frequently upregulated in B-cell lymphomas, and the ectopic expression of miR-17-92 drives B-cell lymphomagenesis in mice 32. In addition, the conditional knock-out of miR-17-92 in c-Myc-driven lymphomas results in increased cell death and reduced tumorigenicity 33. c-Myc also regulates miRNA expression by alternative mechanisms, such as enhancing pri-miRNA processing by directly inducing the expression of DROSHA 34. Liu *et al.* demonstrated that Sophocarpine, a tetracyclic quinolizidine alkaloid, inhibits miR-21 expression by blocking Dicer-mediated miR-21 maturation 35. Hence, c-Myc independent mechanism responsible for miR-21 downregulation by NL101 needs to be further investigated.

Direct therapeutic targeting of c-Myc has been challenging because of its intrinsically disordered structure, which lacks an enzymatic active site for small molecule inhibitors 36. A number of strategies indirectly targeting c-Myc have been reported, which mainly include interfering with c-Myc transcription or translation, promoting c-Myc degradation, and disrupting the c-Myc-Max dimerization 36-38. We describe a novel strategy to inhibit c-Myc by modulating miRNA given that the c-Myc/miRNA circuits contribute to the oncogenic functions of c-Myc. The effect of an individual miRNA on a target's level tends to be subtle, but small changes in the protein level can sometimes have a large physiological effect when a positive feedback loop amplifies the change 39. Indeed, c-Myc directly activates miR-21 transcription, and miR-21 targets Mxd1 to enhance c-Myc transcriptional activity, leading to sustained high levels of miR-21 and c-Myc in aggressive B cell lymphoma (Figure 6A). Through the significant inhibition of miR-21 expression, NL101 efficiently blocks the c-Myc/miR-21/Mxd1 positive feedback loop, which is critical for the survival of B cell lymphoma.

In summary, we demonstrated that NL101 efficiently suppresses the growth of B cell lymphoma *in vitro* and *in vivo*, partly through the induction of apoptosis following DNA damage response. With gene expression profiling, miR-21 was identified as a determinant of NL101 sensitivity in B cell lymphoma. Furthermore, miR-21 represses Mxd1 to enhance c-Myc transcriptional activity, and then c-Myc directly transactivates miR-21 expression. Thus, c-Myc/miR-21/Mxd1 represents a novel positive feedback loop that plays an essential regulatory role in the survival of B cell lymphoma. By targeting miR-21 to block c-Myc/miR-21/Mxd1 axis, NL101 shows its promise as an anticancer agent for B cell lymphoma treatment in the future.
